# Editorial: Depression: social stress, inflammation, neuromodulatory, and neural network perspectives

**DOI:** 10.3389/fpsyg.2025.1605688

**Published:** 2025-04-29

**Authors:** Douglas F. Watt, Daniela Flores Mosri, Simon Boag

**Affiliations:** ^1^9th Dimension Biotech, Nashua, NH, United States; ^2^Department of Psychology, Psychoanalytic Psychotherapy, Neuropsychoanalysis, Universidad Intercontinental, Mexico City, Mexico; ^3^School of Psychology, University of Sydney, Darlington, NSW, Australia

**Keywords:** depression, social stress, inflammation, neuromodulation, neural network

As noted in the precis to this Research Topic, depression is now not only the most expensive condition facing Western societies, but also has become the leading cause of long-term disability, this despite dozens of on- and off-patent mainline antidepressants liberally prescribed (averaging over 9 million scripts per month in the United States alone and a total of 220+ million over 6 years from 2016 to 2022, showing a steady increase year over year). This latter issue, the widening trajectory into disability from depression, appears to have significantly accelerated over the past five decades, and has not been researched in a fashion commensurate with its clinical and public health importance. There remains the worrisome possibility—still not adequately researched empirically—that long-term maintenance on SSRI and SNRI medicines may actually reduce stress resilience and increase vulnerability to relapse and recurrence of depression upon discontinuance, perhaps through promotion of opponent process (via down-regulation of multiple amine systems undergoing long-term perturbation from chronic psychopharmacology). The potential role that mainline antidepressants might have in turning an intermittent-recurrent or relapsing-remitting disorder into a chronic one has been avoided as a research question. In contrast, there is solid if not virtually definitive evidence that psychotherapy of various kinds actually reduces the risk of relapse and recurrence of depression. These concerns about possible major long-term risks associated with chronic antidepressant use are amplified by the clear evidence that these mainline drugs aren't terribly effective antidepressants, barely separating from placebo in the most common form of depression, mild to moderate depression, and where a distinct minority of patients show anything close to remission or a very strong therapeutic response.

All of this might suggest that a default standard of care for the most common forms of depression including most mild-to-moderate depressions should be psychotherapy and social support, and not the reflexive use of SSRI/SNRIs and similar drugs. Unfortunately, the healthcare system in the United States is woefully shorted around adequate mental health care availability and affordability for most individuals, particularly for children and adolescents, where wait lists to see a properly credentialed therapist can be as long as 6 months to a year. Other Western technological societies face very similar difficulties in providing adequate attention to and care for depression. Given its primary mechanistic connections clinically to various forms of social loss, to social defeat, as well as to virtually any type of systemic pro-inflammatory condition, the rising incidence of major depression and its increasingly comorbidity-littered long-term trajectory suggests that there may be many ways in which modern living is “evolutionarily discordant”—that we are asking a still fundamentally hunter-gatherer prosocial genome to live in a radically isolated, sleep deprived, and sedentary fashion, while consuming a pro-inflammatory diet pattern contributing to dysbiosis.

That said, there remain encouraging initiatives in exploring the potential revolutionary impact of ketamine and other psychedelic drugs such as psilocybin, particularly in treatment-refractory depressions, trauma-related depressions with comorbid PTSD, and depressions where suicidality is a significant risk. We still lack empirically derived heuristics to help us understand which types of psychedelic drugs combined with which psychotherapeutic approach or focus might work best. This suggests that depression, with its enormous package of comorbidities in both long-term medical (cancer, cardiovascular disease, neurodegenerative disorders, and type II diabetes) and psychiatric conditions (PTSD, addictions, anxiety disorders, and personality disorders) is still being neglected in public health and public health funding, relative to its very large and worrisome societal impact.

The Research Topic presents a **collection** of papers that cover many different aspects of depression. Guo et al. reported a study to compare psychological disorders and suicide attempts in young population before and after COVID; they showed an increased psychological fragility that highlights depression as the most common driver of fragility/morbidity. Jiang presents two neural hypotheses to explain the negative cognitive bias observed in major depression that involve the interactions between the amygdala, the hippocampus, and the ventromedial prefrontal cortex aiming to contribute to the current models of depression. Zhang et al. highlight the importance of the genetic mechanisms of depression by focusing on gene pathways to investigate the inheritable aspects of depression. Neurochemical potential correlates of depression are explored by Clarici et al., focusing on the role of oxytocin as a mediator of the mother-infant bond, showing that if oxytocinergic signaling is disrupted, mothers are at risk for depression. In terms of supportive treatment for depression with substance use disorder, Durkalec-Michalski et al. conducted research to test the effects of bovine colostrum supplementation showing a decrease in several cytokine levels, the latest evidence that low-grade inflammation may have a role in the etiology of depression. Li et al. consider that there may be a spectrum for depressive disorders; electroencephalographic measurements were used to discern variations in resting-state networks across different depressive state groups. They found significant differences in the frontal and temporal lobes, which may be used as biomarkers for the early detection of depression. In Giacolini et al., the authors proposed a framework for adolescent and young adult depression based on challenges to both two primary independent but interactive emotional systems supporting social bonds and social dominance, with prototype stressors being loss of an important relationship or social bond, vs. a social defeat, competitive loss, loss of status or other dominance reversals. In Hlynsson and Carlbring the authors evaluated and validated the psychometric characteristics, discriminative accuracy, and sensitivity to change of the Generalized Anxiety Disorder 2-item scale (GAD-2) and the Patient Health Questionnaire 2-item scale (PHQ-2) within a clinical population, showing these two measures preserving, with high probability, findings from longer more time consuming and costly psychometric instruments.

To summarize, the increasing prevalence and high risk for both disability and comorbidity associated with depression requires urgent attention. The heavy reliance on antidepressants reflects the still powerful legacy of the “chemical imbalance” meme. Depression is a complex condition with deep connections to how we live and how we relate to others, or fail to, problems that cannot be reduced to brain chemistry alone, and certainly not just to noradrenergic and serotonergic signals impacted by mainline antidepressants. Antidepressants as default choice to treatment allows the many psychosocial determinants of depression such as chronic stress and social disconnection to remain largely ignored. Not only do we need to urgently prioritize mental health funding and to expand access to psychotherapy, but we also need major public health initiatives that address all four of the “pillars of healthy living” (diet, exercise, sleep, social support). Thus, a more holistic, public health-oriented approach—addressing both biological and social-environmental contributors—will be critical to reversing the rising burden of depression in modern society. We remain quite uncertain and ignorant about the real possibility that there are major negative effects on resilience hidden in long-term SSRI and SNRI treatment. We also need to assess the efficacy of alternatives such as psychedelic-assisted therapy, and how psychedelic drugs can be best integrated with what kinds and approaches in psychotherapy to maximize their efficacy and their potential boost to long term emotional resilience.

